# New possibilities of ultrasound in the emergency physician's hands to a young patient with abdominal pain

**DOI:** 10.1186/2036-7902-7-S1-A10

**Published:** 2015-03-09

**Authors:** M Algaba-Montes, A Oviedo-García

**Affiliations:** 1 Emergency Department, Valme Hospital, Seville, Spain

## Background

Abdominal pain is a common symptom in the ER, covering 10% of the assists. The delay in diagnosis and treatment adversely affects the patient's prognosis.Transabdominal ultrasonography is most commonly used to obtain images of hepatobiliary, urogenital, and pelvic structures. However, improvements in ultrasound technology and increasing familiarity with ultrasonographic findings in a variety of gastrointestinal disorders, as Crohn's disease (CD), are broadening its applications, and it is an aspect to be considered by EP in patients with recurrent abdominal pain

## Objective

we present a case of CD, diagnosed at ER, through the use of US scanning used by EP

## Patients and methods

a patient with abdominal pain, with a final diagnosis of an CD

## Results

36 year old male, was admitted to the ER for the third time by abdominal pain. Emergency analytical were unremarkable, as in the preceding cases. Because of the pain the emergency physician underwent an ultrasound scan observing area terminal ileum same transmural thickening with luminal narrowing and decreased peristalsis, involvement of the mesenteric lymph nodes and multiple underlying fat, suspecting CD. We entered the patient performing CT abdomen and ileo-colonoscopy confirmed the diagnosis.

## Conclusion

Bedside ultrasound of the patient by the EP could be an useful tool in cases with abdominial pain whose clinical data and laboratory are unclear. Suspicion of CD, the sensitivity of ultrasound is nearly 90%, especially if ileal location, as in the case presented; being the specific data and the transmural segment thickening, and the presence of fistulae or abscesses. Stenosis exists ultrasound specificity is greater than 95%. Due to its great advantages such as low cost, accessibility, not irradiated and non-invasive ultrasound should be considered in the diagnosis and monitoring of all CD, therefore EP must be trained to diagnose sonographically acute complications of this disease.

## Informed consent

The study was conducted in accordance with the ethical standards dictated by applicable law. Informed consent was obtained from each owner to enrolment in the study and to the inclusion in this article of information that could potentially lead to their identification.
